# Work-Related Risk Assessment According to the Revised NIOSH Lifting Equation: A Preliminary Study Using a Wearable Inertial Sensor and Machine Learning

**DOI:** 10.3390/s21082593

**Published:** 2021-04-07

**Authors:** Leandro Donisi, Giuseppe Cesarelli, Armando Coccia, Monica Panigazzi, Edda Maria Capodaglio, Giovanni D’Addio

**Affiliations:** 1 Department of Advanced Biomedical Sciences, University of Naples Federico II, 80131 Naples, Italy; leandro.donisi@unina.it; 2 Scientific Clinical Institutes ICS Maugeri, 27100 Pavia, Italy; armando.coccia@unina.it (A.C.); monica.panigazzi@icsmaugeri.it (M.P.); edda.capodaglio@icsmaugeri.it (E.M.C.); gianni.daddio@icsmaugeri.it (G.D.); 3 Department of Chemical, Materials and Production Engineering, University of Naples Federico II, 80125 Naples, Italy; 4 Department of Information Technologies and Electrical Engineering, University of Naples Federico II, 80125 Naples, Italy

**Keywords:** biomechanical risk assessment, ergonomics, feature extraction, health monitoring, IMUs, lifting, machine learning, NIOSH, wearable device, work-related musculoskeletal disorders

## Abstract

Many activities may elicit a biomechanical overload. Among these, lifting loads can cause work-related musculoskeletal disorders. Aspiring to improve risk prevention, the National Institute for Occupational Safety and Health (NIOSH) established a methodology for assessing lifting actions by means of a quantitative method based on intensity, duration, frequency and other geometrical characteristics of lifting. In this paper, we explored the machine learning (ML) feasibility to classify biomechanical risk according to the revised NIOSH lifting equation. Acceleration and angular velocity signals were collected using a wearable sensor during lifting tasks performed by seven subjects and further segmented to extract time-domain features: root mean square, minimum, maximum and standard deviation. The features were fed to several ML algorithms. Interesting results were obtained in terms of evaluation metrics for a binary risk/no-risk classification; specifically, the tree-based algorithms reached accuracies greater than 90% and Area under the Receiver operating curve characteristics curves greater than 0.9. In conclusion, this study indicates the proposed combination of features and algorithms represents a valuable approach to automatically classify work activities in two NIOSH risk groups. These data confirm the potential of this methodology to assess the biomechanical risk to which subjects are exposed during their work activity.

## 1. Introduction

Several epidemiological studies provided a strong correlation between physical work exposures and the increased risk of work-related musculoskeletal disorders (MSDs) [1,2,3,4]. Radwin et al. showed the relationship between MSDs and repeated and long durations of external load handling during the workday [5].

Biomechanical exposures during physical work are mainly due to three main factors: intensity (load magnitudes and extent of non-neutral postures), repetition (frequency or number of force exertions and motions) and duration (the time of a physical activity) [6].

In addition to the more traditional quantitative or semiquantitative observational methods [7,8,9,10,11,12,13], occupational ergonomics studies in the field can employ instrumental methods that offer greater agility, precision and duration of measurement. Among the direct measurement methods, wearable inertial systems based on inertial measurement units (IMUs) play an important role in the biomechanical risk assessment [14], and they look very promising for occupational medicine and ergonomics applications [15]. In the field of risk assessment, in fact, wearable inertial technology represents a significant advance in comparison to the evaluation tools traditionally used in ergonomics [16], especially regarding the degree of precision and possibility of automatic measurement detection. IMUs are based on triaxial accelerometers and gyroscopes able to measure 3D acceleration and angular velocity of the sensor with respect to gravity [17]. Often, IMUs also include a triaxial magnetometer useful to give information about the orientation of the sensor in the three-dimensional space. In the absence of standards on the positioning of sensors on the human body [18], the dorsal part of the back was recommended for the ergonomic study of trunk position [19], while the waist was suggested for analysing the overall motion, as representative of centre body mass [20].

In occupational ergonomics, body-worn inertial sensor technology and motion tracking system could be combined to noninvasively collect large amounts of body movement data during physical work [15] and explore their association with occupational risk as assessed with standard methods [21]. The portability and wearability of this technology represents an advantageous alternative to camera-based motion tracking systems [22]. Information on worker exposure obtained through wearable sensors could help to pre-evaluate heavy work, match workers’ skills with physical activity requirements, verify the sustainability of work shift combinations as well as prioritise work modification interventions based on the type and severity of the level of exposure [16]. The success and diffusion of IMUs systems are linked to their relative low cost, the low complexity of the experimental setup and data processing procedures, the limited time constraints and the feasibility of evaluation outside the research laboratories [23]. Several IMUs positioned on the body were used in studies [24,25], where the purpose was to predict, by the same accelerometric data, the geometric (initial and final height, horizontal distance, asymmetry and inclination of the trunk) and temporal (frequency and duration) variables related to lifting, thereby validating the measures that the systems produced.

Among the activities involving biomechanical overloading, material handling and lifting is one of the most studied in the scientific literature, including its association with the development of work-related MSDs [2]. With a view to prevention, NIOSH established a methodology for assessing lifting actions by means of a quantitative method based on intensity, duration and frequency of the task, and other geometrical characteristics of lifting [12]. The method determines the Recommended Weight Limit (RWL) for the lifting tasks and calculates the risk index namely Lifting Index (LI).

In the scientific literature, among the many applications of wearable technology to ergonomics, and in particular among those which use the NIOSH methodology [26], the association of features extracted directly from raw signals (acceleration and angular velocity) with NIOSH risk classes related to repeated load lifting activities has not yet been explored.

Moreover, machine learning (ML) algorithms are gaining popularity in the ergonomic field for biomechanical risk assessment by means of data acquired by wearable inertial systems. Several publications have appeared in recent years documenting several strategies [27,28,29]. IMU systems, which incorporate machine learning into their data analysis pathways, have been found effective in automated exercise detection and in classifying movement quality across a range of lower limb exercises, including lifting, despite studies in this field having so far involved few samples [30].

The question remains whether it is possible to classify lifting tasks belonging to different risk classes according to the value of LI using a machine learning approach by means of features extracted from raw signals.

The aim of this study is twofold: First, we explored the possibility to use a single IMU placed on the lumbar region to monitor the biomechanical risk. Second, we assessed if the time-domain features extracted from acceleration and angular velocity signals acquired by the IMU sensor allowed to classify risk/no-risk tasks according to the NIOSH methodology.

To classify lifting tasks belonging to different LI classes according to the Revised NIOSH Lifting Equation (RNLE), we fed several ML algorithms using time-domain features extracted from acceleration and angular velocity signals. The signals relating to the lifting activities were acquired through the wearable Opal System. The validation of the methodology was carried out through the tenfold cross-validation and different evaluation metrics in order to make the result more robust.

## 2. Materials and Methods

### 2.1. IMU-Based Motion Capture Wearable System: The OPAL System

**Figure 1:**
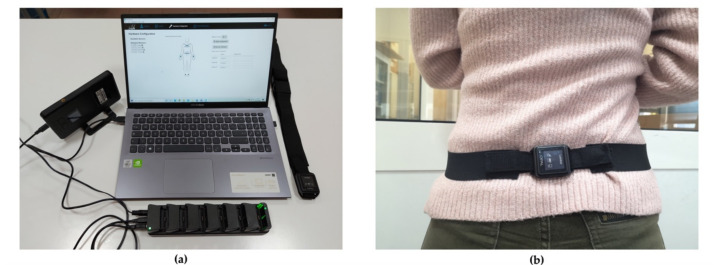
(**a**) Opal System: Access Point, Docking Station, Opal Sensor and Mobility Lab software. (**b**) Placement of the Opal Sensor in lumbosacral region through an elastic belt.

**Figure 2:**
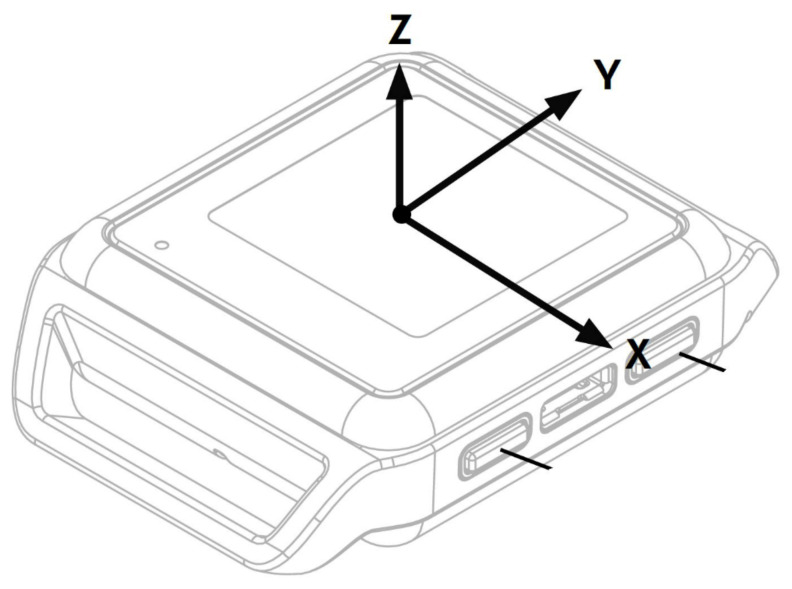
Opal Sensor with the illustration of the *x*-axis, *y*-axis and *z*-axis.

The Opal System by APDM Inc. is the commercial IMU-based wearable system employed in the current study (Figure 1a). The system is composed of several Opal sensors worn by the participants kept in position by straps. Each Opal sensor is composed by a 3-axes accelerometer with 14-bit resolution and two alternative ranges of values (±16 g and ±200 g, where g is the gravitational constant), a 3-axes gyroscope (16-bit, range ± 2000 deg/s) and a 3-axes magnetometer (12-bit, range ± 8 Gauss). The sampling frequency of the acceleration and angular velocity signals was 20 Hz. Opal sensors communicate with a laptop equipped by the Mobility Lab Software by the Bluetooth 3.0. The Access Point manages the communication between the Opal sensors and the laptop, while the Docking Station allows charging and configuring of Opal sensors. The Opal system has proved to be a repeatable [31], reliable [32,33] and accurate [34], and it was used in several scientific studies, e.g., to compute the main spatiotemporal and kinematic parameters relating to the lower limb, upper limb and spine [35,36]. It also allows users to access raw signals such as acceleration and angular velocity. In this study, we used a single Opal sensor positioned on the lumbar region (Figure 1b) [37,38] to acquire raw signals (acceleration and angular velocity) during lifting activities designed to correspond to different risk classes according to the NIOSH methodology. Figure 2 shows the Opal sensor with the direction of the axes.

### 2.2. Revised NIOSH Lifting Equation

The RNLE is a method published by NIOSH to assess the risk of work-related low back disorders (WLBDs) [39]. An equation provides the Recommended Weight Limit (RWL, the weight limit for a healthy worker to safely perform lift tasks during a work shift), starting from a load constant (25–15 kg for the Italian legislation) and through a multiplicative model with six variables relating to the lifting task.

LI, namely the ratio of actual weight to RWL for lifting activity, is a good indicator of the risk of WLBDs for manual lifting [40,41,42].

In the preventive approach, RNLE is useful for verifying that LI is not greater than 1 in lifting activities. Lifting activities with LI values between 1 and 3 are associated with an increased biomechanical risk in the working population, while lifting activities with LI less than 1 indicate acceptable conditions and no risk of WLBDs [12].

The RWL equation is as follows:

RWL = LC × HM × VM × DM × AM × FM × CM(1)

where

- LC: Load Constant = 25/20 kg (males, <45/>45 years old respectively), 20/15 kg (females, <45/>45 years old respectively);
- HM: Horizontal Multiplier;
- VM: Vertical Multiplier;
- DM: Distance Multiplier;
- AM: Asymmetric Multiplier; and
- FM: Frequency Multiplier.

### 2.3. Study Population

Healthy volunteers were recruited from the bioengineering laboratory of the University of Naples “Federico II”. Subjects were included if they were between the ages of 20 and 60, and excluded if they had hypertension (i.e., blood pressure above 100/160 mmHg), or any other heart disease (i.e., congestive heart failure), or a musculoskeletal disorder (i.e., low back pain, herniated disc or trunk surgery). Seven subjects were selected for this preliminary study, whose anthropometric characteristics are shown in Table 1. The study was approved by the local Ethics Committee, in accordance with the Declaration of Helsinki. All participants signed the informed consent.

### 2.4. Study Protocol

Each subject performed a task session based on two trials. Each trial consisted of 30 consecutive lifting tasks. Between the two trials, a pause of 1 h was considered. Specifically, the first trial consisted of repeated liftings in a condition of LI less than one (LI = 0.5) named the NO RISK class, while the second one consisted of repeated liftings in a condition of LI greater than one (LI = 1.3) named the RISK class. LI of 0.5 and 1.3 were derived from the RNLE [12] by variously combining height, frequency and weight of lifting tasks. The details of the combinations are displayed in Table 2. Five out of seven subjects performed the task lifting the load from 50 cm to 125 cm, namely, the optimal geometric condition. One subject performed the task lifting the load from 30 cm to 125 cm, namely, non-optimal geometric conditions (start point: height < 50 cm); one subject performed the task lifting the load from 50 cm to 150 cm, namely, non-optimal geometric conditions (end point: height > 125 cm). The choice of different geometric conditions was adopted to assess the proposed data mining system for the prediction of biomechanical risk in a general context in order to have more interesting and generalisable results.

**Figure 3:**
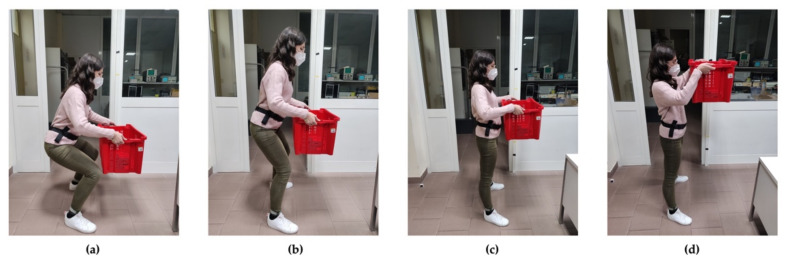
Lifting phases of the lifting task: picking point (**a**) with squatting technique, intermediate points (**b**,**c**) with trunk extension, and destination point (**d**) up to the final height, and then restart the cycle.

The trial (Figure 3) was performed using a plastic container (56 × 35 × 31 cm^3^) with weights equally distributed inside; the size of the plastic container is such that it can be held close to the barycentre of the body during the lifting task. The subjects were instructed to adopt a stable upright posture with the lower limbs slightly apart and to perform the squat technique with a two-handed grip. Each lifting task was performed in a slow, controlled manner without any jerk or sudden acceleration [43]. The researcher was in charge of visually checking the performance, and possibly having the subject repeat the execution to acquire the signal again if the task was carried out in an uncontrolled manner.

### 2.5. Feature Extraction

**Figure 4:**
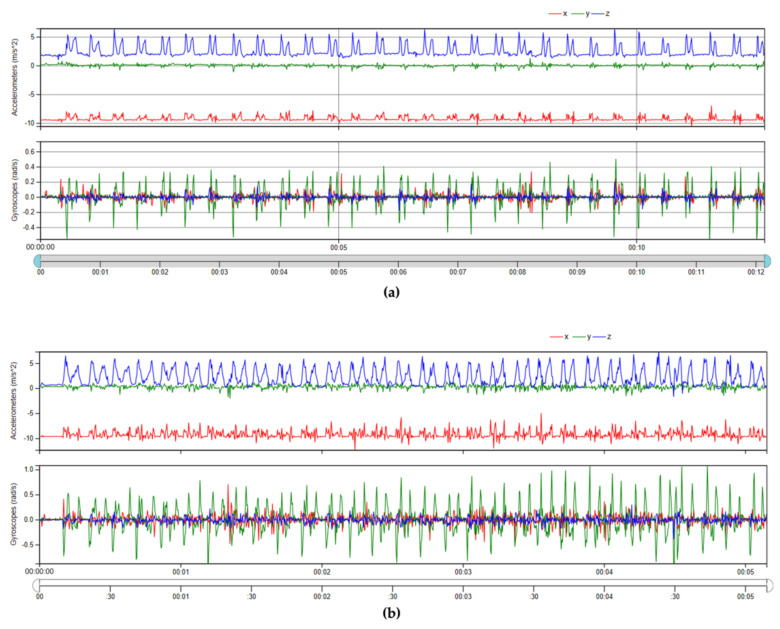
(**a**) Acceleration and angular velocity signals along the 3 axes associated to the NO RISK class, lifting task performed with a LI < 1. (**b**) Acceleration and angular velocity signals along the 3 axes associated to the RISK class, lifting task performed with a LI > 1.

Acceleration and angular velocity signals were not filtered (Figure 4). The signals underwent a segmentation process in order to extract the region of interest (ROI) corresponding to the window time in which the subject performed the lifting task. A temporal synchronisation was performed to pick up the portion of the signal corresponding to the time point of the start and end of the lifting task. For each ROI, several features were extracted in the time-domain. Four features were extracted from each of the three axes of the accelerometer and from each of the three axes of the gyroscope, giving a total of twenty-four attributes. The feature extracted were

- root mean square (RMS),
- standard deviation (SD),
- minimum (MIN) and
- maximum (MAX).

As during the lifting activity the subject moved in two different directions (from bottom to top, and vice versa), giving rise to positive or negative variants of the signals, it was considered that RMS represented the potential differences in the two different study conditions (LI < 1 and LI > 1) better than the average. SD captured the fact that the range of possible acceleration and values of the angular velocity signals can differ for different LI values, as the SD of the acceleration data was associated with the intensity of motion [44]. Both RMS and SD were predictive features in monitoring physical activities [45]. MIN and MAX values were collected as the task was performed in two opposite directions.

### 2.6. Machine Learning and Tools

Several ML algorithms were trained to evaluate the performance of our predictive model based on features in the time-domain extracted from raw signals (acceleration and angular velocity) to discriminate two risk conditions according to the RNLE.

The following ML algorithms were implemented.

Random Forest (RF) is a ML algorithm based on bagging and randomisation. RF uses several decision trees. An instance is assigned to a certain class through a majority vote procedure on the basis of the decision given by the trees that make up the forest. Although this type of ML shows optimal performance on large datasets, it was chosen in this study for its simplicity of parameterisation and for its stability to noise and outliers [46].

Decision Tree (DT) is a hierarchical classifier method, as well as representing the simplest and most used logic-based classification technique [47]. The test data are classified by ordering them as trees based on the values of their characteristics.

Gradient Boost (GB) redefines boosting as a numerical optimisation problem with the goal of minimising model loss of function by adding learning weaknesses and using gradient descent to set the local minimum of the differential function [48]. The GB method implemented in this work is a combination of a decision trees measure and boosting technique.

The AdaBoost (AB) algorithm [49], short for Adaptive Boosting, is a ML meta-algorithm used as an ensemble method. Weights are reassigned to each instance, with higher weights for instances classified incorrectly. Boosting reduces the bias and variance for supervised learning. In the present study, a set of decision stumps (decision trees with only one node and two leaves) was considered.

k-Nearest Neighbour (kNN) ranks the unlabelled instance vector on the basis of the majority label class between its nearest k neighbours in the training set. Several distance metrics, employed to recognise the nearest neighbours, are present in the literature and can influence the algorithm performance. Without prior knowledge, typically the distance metric implemented is the Euclidean distance [50], as in the case under examination. The algorithm usually assumes that the training instances are uniformly distributed across classes, an assumption in line with our perfectly balanced dataset with reference to the two classes. The choice of k has a significant impact on the classification performance [51]; a k equal to 3 was considered in the present work.

Naive Bayes (NB) is a probabilistic ML algorithm, based on Bayes’ Theorem. It calculates the probability of each class for a specified instance and then returns the class with the highest probability. This algorithm, which requires little data for training and little storage space, is suitable for the small size of the datasets that perform the analysis for each subject in our study. It is also fast during training without many parameters to set, based on the assumption of the conditional independence of the characteristics [52].

Multilayer Perceptron (MLP) consists of multiple layers of simple, two-state sigmoid processing elements, i.e., nodes or neurons interacting using weighted connections, only between neurons of adjacent layers [53]. In the present study, a configuration with a single hidden layer and 10 neurons was considered.

Support Vector Machine (SVM) in a binary classification, as in the case under study, creates a hyperplane that separates data from two different classes. The largest possible distance is established between the separating hyperplane by maximising the margin, thus creating the separation [54]. The kernel choice determines the separation boundary of the classes. The Radial Basis Function (RBF) or Gaussian Kernels are the most popular kernels used as default for any nonlinear model, but also polynomial kernels are very popular [55]. In our study, we used SVM with a linear kernel.

Logistic Regression (LR) is an efficient and powerful way to analyse the effect of several independent variables on a binary outcome, as in the case under study, and allows quantifying the contribution of each feature. LR iteratively identifies the strongest linear combination of variables with the highest probability to detect the observed outcome [56].

K-fold cross-validation is one of the most widely used approaches for estimating classifier error and was employed in our study as tenfold cross-validation (CV) to validate the predictive models described above and provide more robust evidence on our predictive biomechanical risk model, based on features extracted from raw signals. Tenfold CV involves splitting a dataset into ten subsets, with the iterative use of nine to train the model and one to evaluate its performance [57]. In this work, a stratified CV was adopted in order to keep the proportions between the two classes unaltered among the folds [58].

Moreover, in order to better generalise the validation of our proposed approach, we used a leave-one-subject-out cross-validation using six subjects to train the predictive models and one subject to test the algorithms.

The performance of the predictive models proposed was evaluated through the following evaluation metrics: Accuracy, Sensitivity, Specificity and Area under the curve Receiver operator characteristic (AucRoc). The Accuracy metric is a measure of the ratio of correct predictions over the total number of instances considered. The Sensitivity metric is employed to measure the fraction of positive patterns that are correctly classified, while the Specificity metric is used to measure the fraction of negative patterns that are correctly classified. Finally, the AucRoc reveals the generally ranking performance of a classification algorithm.

The Confusion Matrix of the best algorithm in terms of evaluation metrics scores was also reported, with matching between instances in an expected class and an actual class [59].

Finally, the Feature Importance was also reported through the calculation of the Information Gain (IG), an indicator of the amount of information provided by the features [60]. A feature selection on the basis of the IG was implemented as filter method before the classification task. WEKA data mining software was used for the feature importance analysis [61], selecting InfoGainAttributeEval as Attribute Evaluator and Ranker as Search Method.

ML algorithms have been implemented through the artificial intelligence platform Knime Analytics Platform (version 3.7.1), which finds increasing diffusion in the scientific literature [62,63].

## 3. Results

First, we performed a ML analysis for each subject to assess the feasibility of the proposed data mining system to assess the biomechanical risk for a single subject. For each subject, we considered two datasets: the first dataset is composed of 60 instances, 30 for each class (NO RISK, RISK), and 12 features extracted from the acceleration signals; the second dataset is composed of 60 instances, 30 for each class (NO RISK, RISK), and 12 features extracted from the angular velocity signals. For each dataset, we performed the ML analysis by averaging the results among the seven subjects, and we further showed the standard deviation in order to include prediction uncertainty. The results for each dataset are shown in Table 3 and Table 4, respectively, where Sensitivity and Specificity were computed considered as reference for the NO RISK class.

**Figure 5:**
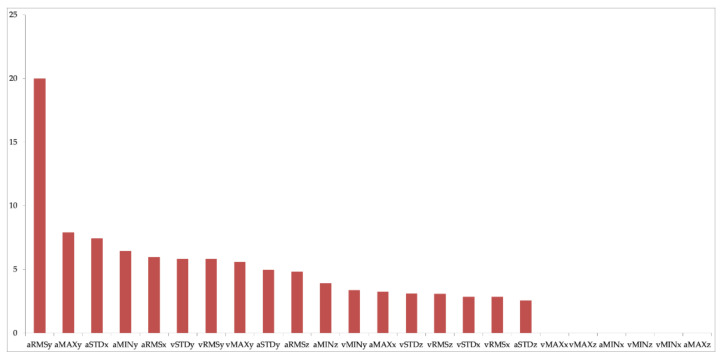
Feature importance based on the Information Gain value. Abbreviations. aRMSx: *x*-axis acceleration Root Mean Square; aRMSy: *y*-axis acceleration Root Mean Square; aRMSz: *z*-axis acceleration Root Mean Square; aSTDx: *x*-axis acceleration Standard Deviation; aSTDy: *y*-axis acceleration Standard Deviation; aSTDz: *z*-axis acceleration Standard Deviation; aMINx: *x*-axis acceleration Minimum; aMINy: *y*-axis acceleration Minimum; aMINz: *z*-axis acceleration Minimum; aMAXx: *x*-axis acceleration Maximum; aMAXy: *y*-axis acceleration Maximum; aMAXz: *z*-axis acceleration Maximum; vRMSx: *x*-axis angular velocity Root Mean Square; vRMSy: *y*-axis angular velocity Root Mean Square; vRMSz: *z*-axis angular velocity Root Mean Square; vSTDx: *x*-axis angular velocity Standard Deviation; vSTDy: *y*-axis angular velocity Standard Deviation; vSTDz: *z*-axis angular velocity Standard Deviation; vMINx: *x*-axis angular velocity Minimum; vMINy: *y*-axis angular velocity Minimum; vMINz: *z*-axis angular velocity Minimum; vMAXx: *x*-axis angular velocity Maximum; vMAXy: *y*-axis angular velocity Maximum; vMAXz: *z*-axis angular velocity Maximum.

Second, we performed Feature Importance by means of the calculation of the IG, considering the entire study sample and the features extracted from both the acceleration and angular velocity signals along the three axes (Figure 5).

Third, we performed a ML analysis considering all seven subjects to assess the feasibility of the proposed data mining system for biomechanical risk assessment for a general study population. In this analysis, we considered a unique dataset consisting of 420 (60 × 7) instances, 210 for each class (NO RISK, RISK), and 18 features extracted from both acceleration and angular velocity signals excluding the six features with IG equal to zero (Figure 5). In our study, the general rule is respected that foresees at least equal to 10 the ratio n/d, between the number n of instances available in the training set and the dimension d of the feature space [64]. This strengthens and makes the result of our analysis shareable.

The results of the ML analysis on the entire dataset are shown in Table 5, where the NO RISK class was considered as the reference class for Sensitivity and Specificity.

Table 6 shows the Confusion Matrix of the best algorithm (GB) resulting from the analysis on the entire study sample and according to the scores of the evaluation metrics: Accuracy, Sensitivity, Specificity and AucRoc. The resulting confusion matrix is perfectly balanced with the following values: TP = 197, FP = 13, FN = 8, TN = 202.

Finally, we performed a ML analysis on the entire dataset using as validation strategy the leave-one-subject-out, namely, using six subjects for the training set and one subject for the test set. Results are shown in the Table 7.

## 4. Discussion

The goal of our research was to explore the feasibility of several state-of-the-art ML algorithms—fed with specific time-domain features extracted from the acceleration and angular velocities signals during a lifting activity—to classify the lifting risk classes based on the LI values according to the RNLE. The results obtained suggest ML algorithms—operating on the time-domain features (RMS, SD, MIN and MAX) extracted during lifting activities from the acceleration and angular velocity signals along the three dimensions of space—can offer valid help to experts in ergonomics for the precise and automatic classification of the biomechanical risk of workers engaged in load-lifting activities.

The ML analysis performed was aimed at the classification of lifting activities based on the presence or absence of risk, defined by the LI index.

First, having carried out a ML analysis for each subject, we obtained the average scores and the standard deviations of the evaluation metrics of the seven subjects by considering separately the characteristics extracted from the acceleration signal and the angular velocity signal. As shown in Table 3 and Table 4, the application of state-of-the-art algorithms on the time-domain features extracted from the acceleration signal provides superior performance (evaluation metric scores) compared to that achieved by using the angular velocity signal, albeit with satisfactory results for the latter as well. The proposed combination of algorithms and features extracted from the acceleration signal achieved an accuracy of between 0.79 and 0.98, a sensitivity between 0.79 and 0.98, a specificity between 0.79 and 0.99, and AucRoc between 0.84 and 0.99. The proposed combination of algorithms and features extracted from the angular velocity signal achieved an accuracy between 0.68 and 0.90, a sensitivity between 0.84 and 0.91, a specificity between 0.44 and 0.92, and an AucRoc score between 0.82 and 0.94.

Second, in our study, eighteen out of twenty-five features showed a non-zero IG (Figure 5), highlighting their predictive power for this specific classification task. Specifically, the most informative features, according to IG, were those associated with the acceleration of the *y* axis, i.e., the mediolateral direction (Figure 2). This means that the trajectory of the subject’s centre of gravity along the *y* axis during the lifting task (Figure 3) tends to have a greater information to separate risk classes, despite the load being moved along the x trajectory. In particular, the aRMSy alone shows an information gain equal to 20%, highlighting its high discriminating power between the two risk conditions.

As for the features relating to the acceleration along the *y* axis, the most informative ones according to IG (Figure 5) are, once again, those relating to the angular velocity around the *y* axis. Approximately 60% of the information provided by the features derives from those relating to the angular velocity and acceleration of the *y* axis, and this result must be taken into account.

Third, as shown in Table 5, we performed a ML analysis considering the whole sample of the study in order to have more generalisable results and to evaluate if the proposed method was applicable not only to the single subject, but also to a whole sample. This property could in fact represent a substantial advantage for using the proposed methodology during preventive interventions for the health and safety of workers in the workplace. This further analysis was carried out using the features extracted from both acceleration and angular velocity signals relating to the three axes (considering the entire study sample, and excluding the features with IG equal to zero). All classifiers (with the exception of the NB, SVM and LR algorithms) showed an accuracy between 0.80 and 0.95, a sensitivity between 0.72 and 0.94, a specificity between 0.85 and 0.96, and AucRoc between 0.90 and 0.99. As shown in Table 5, six out of nine ML algorithms discriminated excellently (AucRoc values > 0.90) the two risk classes. Conventionally, AucRoc values > 0.70 are considered to represent moderate discrimination, value > 0.80 good discrimination and values > 0.90 excellent discrimination; on the basis of the results, as shown in the Table 5, six of nine ML algorithms showed an excellent discrimination of the two risk classes. The poor performance of the NB algorithm was due to the presence of a statistically significant correlation between characteristics [56] (correlation study not shown). The LR algorithm, as the NB one, is based on the concept of probability, and this could explain the limited performances of LR. Instead, the poor performance of the SVM with linear kernel could be explained by the fact data are not linearly separable. The best algorithm was the GB which reached values of 0.95, 0.94, 0.96 and 0.99 in Accuracy, Sensitivity, Specificity and AucRoc, respectively. As shown in Table 6, the almost symmetric Confusion Matrix of GB presents only 21 instances wrongly classified out of 240 total instances, confirming the potential of this methodology applied to biomechanical evaluation.

Finally, to better generalise the performance of our models, we tested them using leave-one-subject-out, training the classifiers on six subjects and testing them on one subject. Although the metrics resulted slightly lower, the data shown in Table 7 are comparable with the ones obtained from the stratified tenfold CV. Once again, the tree-based ML algorithms has proven more efficient in terms of evaluation metrics for this purpose.

This is the first study that considers risk discrimination (by ML) according to NIOSH using a single IMU placed on the subject’s pelvis to extract four basic time-domain features. The achieved results, when compared with the recent ones described by other research groups, are in line or superior to those based on more complex methodologies.

In the study by Varecchia et al. [65], the combination of an artificial neural network fed with time-domain and frequency-domain features extracted from surface electromyography and optoelectronic systems resulted in classification Accuracy of up to 90% against three NIOSH risk classes (LI = 1, LI = 2, LI = 3). In a subsequent work by the same authors [66], the new feature of Lifting Energy Consumption [67] was used to feed a neural network similar to the previous one, demonstrating an Accuracy up to 100%. The limit of this methodology, as pointed out by the authors themselves, is due to the poor applicability in the workplace, an aspect that is solved using wearable inertial sensors as in our case.

Snyder et al. [68,69] proposed a modified Convolutional Neural Network model to distinguish three risk levels (low, medium and high) according to the American Conference of Governmental Industrial Hygienist Threshold Limit Values for lifting. Similar to our work, they used IMU sensors, albeit in larger number, to achieve 90% Accuracy.

With a similar goal to ours, Brandt et al. [43] tried to classify lifting activities into low- and high-risk categories according to the guidelines of the Danish Working Environment Authority, reaching an Accuracy score equal to 65% using a Linear Discriminant Analysis algorithm. In the study by Conforti et al. [27], which aimed to distinguish between correct and incorrect postures, the extraction of data from an IMU positioned on the pelvis of the subject, and coupled with an IMU placed on the trunk, did not allow obtaining scores higher than 75% using a Support Vector Machines algorithm with four different kernels.

Although the presented methodology is powerful, doubts could be raised about the effective capabilities of a single IMU for the validation of such results. Although a single IMU on the pelvis is not sufficient to fully predict the parameters associated with lifting (e.g., the weight of the object to be handled, the horizontal distance, etc.), this solution estimates the lumbar load fairly well, when the displaced mass is known and is in a consistent position with respect to the body [38]. In addition to significantly increasing the convenience in field trials, the use of a single IMU, positioned on the back, is considered sufficient to provide the data necessary to distinguish lifting classes [68].

Based on these results, the experimented approach—which combines time-domain features and machine learning algorithms—proved to be a valid indicator, although preliminary because of the low number of samples analysed, of the risk of WLBDs for manual lifting (according to the NIOSH index) to which workers are potentially exposed during their working activity.

## 5. Conclusions

In conclusion, the results showed that the proposed combinations of features—extracted from acceleration and angular velocity signals acquired by a single IMU placed at pelvis—and state-of-the-art ML algorithms represents a potential valid approach to automatically classify the biomechanical risk to which subjects may be exposed during lifting activities, for example, at work. The presented methodology could represent a valid integration to the established protocols (e.g., NIOSH lifting equation) to evaluate the biomechanical risk more quickly and easily. Moreover, it could represent a valid alternative when the conditions required for the application of standardised evaluation methods do not exist. These results are of direct practical relevance for occupational ergonomics, as they present the opportunity for automatic, economic and non-invasive detection (by placing an IMU on the pelvis of the subjects), to assess the musculoskeletal and biomechanical risk associated with lifting. Based on the promising results presented in this article, the next phase of our research could consider an extension of the sample under consideration to further validate the methodology, new experiments that consider more than two LI values, and the integration of additional features from the time-domain and possibly the frequency-domain. The main limitation of the study is in fact the low sample size making this a preliminary study. Future work will benefit both from the inclusion of larger groups of participants as well as the use of deep learning techniques for classification such as convolutional neural networks [70]. It is known these are especially advantageous for analysing time series data, reducing the risk of overfitting and improving system Accuracy.

## Figures and Tables

**Table 1:** Anthropometric characteristics of the study population presented as mean ± standard deviation.

Age (years)	27.71	±	1.60
Height (cm)	167.40	±	4.86
Weight (kg)	69.00	±	69.00
Body Mass Index (kg/m^2^)	24.51	±	24.51

**Table 2:** Combinations of the height, frequency and weight variables for lifting activities corresponding to LI 0.5 and 1.3.

First Trial LI < 1 (0.5)				Second Trial LI > 1 (1.3)				
Displacement [Start–End] (cm)	Frequency (Lifts/min)	Weight Lifted (kg)		Displacement [Start–End] (cm)	Frequency (Lifts/min)		Weight Lifted (kg)	
	m & f	m	f		m	f	m	f
[50–125]	2.5	7	5	[50–125]	6	4	15	10
[30–125]	2.5	5	4	[30–125]	5	3	13	8
[50–150]	2.5	5	4	[50–150]	5	3	13	8

**Table 3:** Scores from stratified tenfold cross-validation (CV) evaluation metrics averaged over the seven subjects (mean ± standard deviation) using the features extracted from the acceleration signals along the three axes.

Algorithms	Accuracy	Sensitivity	Specificity	AucRoc
DT	0.97 ± 0.05	0.97 ± 0.04	0.97 ± 0.05	0.97 ± 0.03
RF	0.98 ± 0.02	0.98 ± 0.03	0.99 ± 0.02	0.99 ± 0.01
GB	0.97 ± 0.04	0.98 ± 0.03	0.96 ± 0.06	0.98 ± 0.02
AB	0.98 ± 0.02	0.98 ± 0.02	0.98 ± 0.04	0.97 ± 0.03
kNN	0.90 ± 0.09	0.88 ± 0.10	0.91 ± 0.11	0.94 ± 0.06
NB	0.96 ± 0.03	0.96 ± 0.04	0.96 ± 0.04	0.99 ± 0.01
MLP	0.95 ± 0.06	0.93 ± 0.07	0.96 ± 0.06	0.97 ± 0.03
SVM	0.83 ± 0.20	0.83 ± 0.29	0.82 ± 0.37	0.85 ± 0.22
LR	0.79 ± 0.15	0.79 ± 0.14	0.79 ± 0.19	0.84 ± 0.13

Abbreviations. AB: AdaBoost; AucRoc: Area under the curve Receiver operator characteristic; DT: Decision Tree; GB: Gradient Boost; kNN: k-Nearest Neighbor; LR: Logistic Regression; NB: Naïve Bayes; MLP: Multilayer Perceptron; RF: Random Forest; SVM: Support Vector Machine.

**Table 4:** Scores from stratified tenfold CV evaluation metrics averaged over the seven subjects (mean ± standard deviation) using the features extracted from the angular velocity signals along the three axes.

Algorithms	Accuracy	Sensitivity	Specificity	AucRoc
DT	0.88 ± 0.11	0.87 ± 0.14	0.88 ± 0.08	0.88 ± 0.12
RF	0.90 ± 0.11	0.90 ± 0.13	0.89 ± 0.09	0.94 ± 0.10
GB	0.89 ± 0.13	0.90 ± 0.14	0.88 ± 0.13	0.92 ± 0.09
AB	0.89 ± 0.14	0.89 ± 0.16	0.89 ± 0.12	0.92 ± 0.12
kNN	0.82 ± 0.10	0.81 ± 0.14	0.82 ± 0.09	0.88 ± 0.09
NB	0.86 ± 0.12	0.83 ± 0.17	0.89 ± 0.08	0.92 ± 0.08
MLP	0.90 ± 0.12	0.88 ± 0.16	0.92 ± 0.09	0.94 ± 0.08
SVM	0.68 ± 0.19	0.91 ± 0.13	0.44 ± 0.42	0.82 ± 0.16
LR	0.84 ± 0.08	0.84 ± 0.13	0.84 ± 0.05	0.90 ± 0.07

Abbreviations. AB: AdaBoost; AucRoc: Area under the curve Receiver operator characteristic; DT: Decision Tree; GB: Gradient Boost; kNN: k-Nearest Neighbor; LR: Logistic Regression; NB: Naïve Bayes; MLP: Multilayer Perceptron; RF: Random Forest; SVM: Support Vector Machine.

**Table 5:** Scores from stratified tenfold CV evaluation metrics considering the entire study sample and using the features extracted from both acceleration and angular velocity signals along the three axes with a non-zero IG.

Algorithms	Accuracy	Sensitivity	Specificity	AucRoc
DT	0.91	0.89	0.92	0.93
RF	0.95	0.94	0.95	0.99
GB	0.95	0.94	0.96	0.99
AB	0.80	0.72	0.87	0.90
kNN	0.84	0.83	0.85	0.91
NB	0.67	0.63	0.71	0.75
MLP	0.91	0.91	0.91	0.97
SVM	0.71	0.70	0.71	0.79
LR	0.66	0.67	0.66	0.68

Abbreviations. AB: AdaBoost; AucRoc: Area under the curve Receiver operator characteristic; DT: Decision Tree; GB: Gradient Boost; kNN: k-Nearest Neighbor; LR: Logistic Regression; NB: Naïve Bayes; MLP: Multilayer Perceptron; RF: Random Forest; SVM: Support Vector Machine.

**Table 6:** Confusion matrix of the best algorithm in terms of evaluation metrics scores: the GB.

	NO RISK	YES RISK
NO RISK	197	13
YES RISK	8	202

**Table 7:** Scores from leave-one-subject-out evaluation metrics considering the entire study sample and using the features extracted from both acceleration and angular velocity signals along the three axes.

Algorithms	Accuracy	Sensitivity	Specificity	AucRoc
DT	0.88	0.93	0.83	0.88
RF	0.88	0.97	0.80	0.97
GB	0.75	0.97	0.53	0.92
AB	0.88	0.97	0.80	0.96
kNN	0.50	0.00	1.00	0.48
NB	0.82	0.97	0.67	0.97
MLP	0.73	0.97	0.50	0.87
SVM	0.77	0.97	0.57	0.82
LR	0.70	0.97	0.43	0.72

Abbreviations. AB: AdaBoost; AucRoc: Area under the curve Receiver operator characteristic; DT: Decision Tree; GB: Gradient Boost; kNN: k-Nearest Neighbor; LR: Logistic Regression; NB: Naïve Bayes; MLP: Multilayer Perceptron; RF: Random Forest; SVM: Support Vector Machine.

## Data Availability

The datasets generated and/or analysed during the current study are not publicly available due to privacy policy but are available from the corresponding author on reasonable request.
